# Energy metabolism disturbance, altered neuronal development and glutamatergic signalling in human derived neuronal cell models of ADHD

**DOI:** 10.1192/j.eurpsy.2024.87

**Published:** 2024-08-27

**Authors:** S. D. Kittel-Schneider, AG McNeill/Kittel-Schneider

**Affiliations:** ^1^Department of Psychiatry and Neurobehavioural Science, University College Cork, Cork, Ireland; ^2^Department of Psychiatry, Psychotherapy and Psychosomatic Medicine, University Hospital of Wuerzburg, Wuerzburg, Germany

## Abstract

**Abstract:**

Despite major advances in research into the neurobiological basis of mental illness, there have been hardly any new developments in new drug therapies. As there are approximately 30% of affected individuals that do not respond sufficiently to available treatments, there is a significant unmet medical need for new therapeutic approaches. About 90% of novel substances that have shown promise in animal studies are not effective in clinical trials. Recent research on human induced pluripotent stem cells (hiPSC) could lead to the use of more human-tailored models in this field. IPSC-derived cell models and organoids may be very attractive for preclinical screening and bridge the gap between in vitro and in vivo studies, reducing animal testing. However, the next steps must first demonstrate the validity and reproducibility of the initial functional results from the hIPSC models of mental illness. In our own studies on neuronal cell models of patients with attention-deficit/hyperactivity disorder (ADHD) with rare PARK2 gene variants, we were able to show evidence of mitochondrial dysfunction and impaired energy metabolism. Additionally, we have first hints at a oxidative dysbalance which could be as well targeted by medication. In a model of cortical development of ADHD patients with common variants in the ADGRL3 gene, we found first evidence for altered neuronal maturation as well as abnormalities in calcium metabolism and glutamatergic functionality compared to cells from healthy controls. In summary, these first results are promising that hIPSC models can contribute new insights into cellular pathomechanisms of mental and neurodevelopmental disorders and the development of new, individualised therapeutic approaches.

**Disclosure of Interest:**

S. Kittel-Schneider Grant / Research support from: The studies are funded by IZKF Wuerzburg and BBRF fund (to SKS and RMcN). SKS received speaker’s honoraria from Takeda, Medice and Janssen.

